# Assessment of the feasibility of a community-based mental health training programme for persons with disabilities by non-specialists from different stakeholders’ perspectives in Bangladesh

**DOI:** 10.1186/s12913-024-10742-5

**Published:** 2024-03-04

**Authors:** Kamrun Nahar Koly, Jobaida Saba, Enryka Christopher, Anan Nisat Nabela Hossain, Taslima Akter, Zakia Rahman, Helal Uddin Ahmed, Julian Eaton

**Affiliations:** 1 Health System and Population Studies Division, International Centre for Diarrhoeal Disease Research, Bangladesh (icddr,b), Mohakhali, Dhaka, 1212 Bangladesh; 2 Trauma and Community Resilience Center, Boston Children’s Hospital & Harvard Medical School, Boston, Massachusetts United States of America; 3 Centre for Disability in Development, Dhaka, Bangladesh; 4 CBM Bangladesh Country Office (CBMBCO), Dhaka, Bangladesh; 5 National Institute of Mental Health, Dhaka, Bangladesh; 6 Centre for Global Mental Health, London School of Hygiene & Tropical Medicine, London, United Kingdom; 7 CBM Global Disability and Inclusion, Laudenbach, Germany

**Keywords:** Mental health, Person with disabilities, Peer support, Training programme, Bangladesh

## Abstract

**Introduction:**

Bangladesh is a developing country where 11% of the population has at least one disability, but no community-level mental health service is available. There is limited evidence of the burden of mental health issues and health-seeking behaviour among this population. This study assessed the feasibility of a training intervention for persons with disabilities, where peer support providers provided community-based disability-inclusive mental health services.

**Methods:**

Four stakeholder groups participated in this qualitative study: peer responders (trained persons with disabilities), trainers, representatives of organisations of persons with disabilities and disability-specific organisations, and officials of international and national non-governmental organisations. Two types of qualitative interviews were used to collect data, and thematic analysis techniques were utilised.

**Result:**

Stakeholders perceived the peer responder training programme as acceptable for persons with disabilities to develop themselves as peer support providers, with potential benefits including increased mental health literacy, ensuring accessible mental health services, and improving the well-being of persons with disabilities. Potential challenges included receiving training and delivering services. Increased training duration, more fieldwork, supervision opportunities, and refresher training were recommended to mitigate training challenges. Financial support and formal community recognition were deemed necessary for training delivery.

**Conclusion:**

The peer responder training programme was feasible to ensure accessible mental health services for persons with disabilities, build a workforce to screen for mental health conditions, and provide appropriate referrals. A multi-sectoral collaboration of government and non-governmental institutions is recommended to policy advocates to expand the peer responder training programme in the mainstream mental healthcare system.

**Supplementary Information:**

The online version contains supplementary material available at 10.1186/s12913-024-10742-5.

## Introduction

Bangladesh is a low- and middle-income country (LMIC) with a population of over 160 million. The prevalence of mental health (MH) conditions in Bangladesh ranges from 6.5 to 31% [1]. Despite this high prevalence, only a few MH service providers (250 psychiatrists and 500 psychologists) exist nationwide, concentrated in urban areas [2, 3]. MH-related stigma and lack of information also negatively impact those affected [3]. Vulnerable groups, like people with disabilities, face additional challenges accessing MH services [4–7]. An estimated 18 million people live with a disability in Bangladesh [8]. However, due to a lack of research, the prevalence of MH conditions in those with disabilities in Bangladesh is still unknown.

According to the United Nations Convention on the Rights of Persons with Disabilities (UNCRPD), persons with disabilities are associated with long-term physical, mental, intellectual, or sensory impairments. These impairments interact with societal barriers, hindering full and effective social participation. Global studies on persons with hearing or visual impairment and limb disabilities indicated above-average MH conditions such as depression (11.4–84%), anxiety (3.4–10%), post-traumatic stress disorder (PTSD) (3.3–56.3%), and psychological distress (31.7%) [9–22]. Physical impairments can have detrimental effects on quality of life (QoL) due to reduced social interaction, financial strain, social stigma, poor body image, or low self-esteem [9–22]. Additionally, the COVID-19 pandemic increased stressors like disruption in accessing healthcare services, financial and food insecurity, isolation, loneliness, interrupted daily routines, and fear of infection, further worsening MH in vulnerable populations [23–25].

Poor access to health information and healthcare services is a significant barrier to seeking care among people with disabilities [26, 27]. Countries have identified strategies to improve access, such as developing an alternative non-specialist workforce to provide services and information to households [28]. An inadequate MH workforce is a major challenge in the health systems of most LMICs [29]. Consequently, low identification, treatment delay, and lack of awareness of MH conditions are common in these nations [29]. In Bangladesh, MH-seeking behaviour is notably low (12%), with a 92.3% treatment gap in the overall population, higher than in neighbouring India, Pakistan, and Myanmar [30–32]. There is no conclusive research on the MH-seeking behaviour of persons with disabilities in Bangladesh.

A task-sharing approach involving non-specialists in MH service provision through training and supervision can address unmet MH needs in low-resource areas [33]. In many countries, non-specialists, such as peers with comparable socio-cultural, linguistic, and life experiences, deliver community-level MH support within and outside existing health systems [34–37]. In several studies, peers have provided psycho-social and psycho-therapeutic interventions for different populations in LMICs [38–41]. MH services through peer support programmes have shown a positive effect on mental well-being, QoL, empowerment, self-management skills, and treatment adherence in groups such as persons with severe mental illnesses, young adolescents, older adults, substance users, and persons with physical disabilities [40, 42–46]. An Iranian study reported a significant association between peer support and subjective well-being among visually impaired adolescents [36]. In addition, a systematic review suggested that peer support could bridge MH disparities and treatment gaps by reaching out to underserved, disadvantaged groups [47].

A similar approach was adopted in Bangladesh by the Centre for Disability in Development (CDD), with support from the United Nations Economic and Social Commission for Asia and the Pacific (UNESCAP), to protect and empower people with disabilities during the COVID-19 pandemic [48]. CDD is one of the largest national organisations that advocate for disability rights. They implemented a peer responder training (PRT) programme to promote community-based, inclusive MH services for people with disabilities in Bangladesh. This PRT programme trained peer responders who are also persons with disabilities with lived experiences of mental health conditions to improve access to community-based support for MH needs. There is limited evidence about the acceptability and feasibility of peer-mediated MH support, particularly for people with physical and sensory impairments in low-income settings, especially in South Asia. Such research is crucial for generating opportunity and momentum for changes in practice, expanding access to services, and improving outcomes for MH conditions. This study aimed to assess the feasibility of this community-based inclusive MH PRT programme from different stakeholders’ perspectives in Bangladesh.

## Methodology

This qualitative descriptive study analyses interviews and focus group discussions with peer responders (trained persons with disabilities); trainers (psychologists); members of organisations of persons with disabilities (OPDs), disability-specific organisations (DSOs), non-governmental organisations (NGOs), and international NGOs [49].

### Ethical considerations

Guidelines for research from the National Disability Authority (NDA) and the UNCRPD were followed [50, 51]. As this study included a vulnerable population, clear information about the study was provided, and verbal and written consent was obtained before the interviews. The Institutional Review Board (IRB) of icddr, b approved the study (PR 22079).

### Study settings

The PRT programme falls under the CDD’s umbrella project entitled “Protecting and Empowering Persons with Disabilities in the context of the COVID-19 Pandemic **(**PEPD COVID-19)**.**” This 15-month project was implemented in 10 catchment areas in Dhaka and Chattogram through six OPDs and DSOs from March 2021 – May 2022. Dhaka is the capital of Bangladesh, and Chittagong is the second-largest city in the country. The peer responders went through PRT training from 5th September to 9th September 2021 and implemented their training in the community from 12th September 2021 to 31st May 2022. Refresher training was conducted 5 months after the first session of the training. The study was conducted from 5th August to 31st October 2022. Participants were interviewed face-to-face or online based on their preferences and availability.

### Peer responder training (PRT) Programme

The programme was developed based on findings from the "Promotion of Human Rights of Persons with Disabilities in Bangladesh (PHRPBD)" programme facilitated by CDD with funding support from the CBM Global Disability Inclusion [52]. Project-related information is published elsewhere [53]. Findings from PHRPBD suggested that self-help groups could effectively promote empowerment and peer-to-peer psycho-social support. Therefore, the CDD, which primarily works with persons with physical disabilities, speech, visual and hearing impairments, developed a PRT programme at the height of COVID-19 outbreaks to train those with physical disabilities and visual impairments as peer responders in some selected communities. The term “peer” is characterized by people who share similar experiences and may relate to one another easily, and thus provide more sincere empathy and affirmation [54]. Therefore, in the context of the peer responder training module, the term “peer” means persons with disabilities who might or might not have mental health challenges. The reason for involving these persons with disabilities was that they had similar socio-cultural backgrounds and life experiences as the other people with disabilities in their communities. Having similar experiences would eventually help them to connect with their peers and empathise. The peer responders training programme was a pilot intervention project that intervened among persons with physical disabilities and visual impairments. These peer responders were open to providing mental health support to other persons with physical disabilities, speech, hearing, or visual impairments. As they went through multiple levels of the capacity-building process through different CDD projects, such as “Enhancing Community Based Inclusive Development (ECBID)”, they were able to communicate with persons with hearing disabilities. Also, they were accompanied by supportive team members for using sign-language with persons with hearing impairments. Moreover, another inclusion criterion was people with disabilities with secondary or higher education. All participants were affiliated with OPDs and DSOs in their respective communities.

An extensive literature review, alongside the formation of an expert panel, was done to develop an informed and comprehensive intervention. The panel comprised psychiatrists, clinical psychologists, and other professionals with experience in the disability sector. Furthermore, people with disabilities and their caregivers, especially those who sought help from CDD due to a prior history of MH issues, were involved in the intervention’s design. Training and fieldwork started in September 2021. Refresher training occurred 5 months after the first training session. Programme content is outlined in Appendix A.

### PRT programme activities

#### Phase 1: Capacity building and empowering peer responders

Thirty participants were trained as peer responders over 5 days. Training included three broad modules across 15 chapters. Module 1, “Understanding basic mental health,” comprised discussions about what MH is and the symptoms of different MH conditions. Peer responders were introduced to the concept of psychometric tools and trained on administering a few of them, including DASS-21 (Depression, Anxiety, and Stress Scale), GAD-7 (Generalised Anxiety Disorder), PHQ-9 (Patient Health Questionnaire), and World Health Organization (WHO-5) Well-being Index. All these tools were previously adapted, simplified and validated in different mental health research. Moreover, peer responders were engaged with CDD in other livelihood projects for a long time, where they were trained to use these tools for assessing the well-being of persons with disabilities. In order to guide peer responders about the psychometric tools, trainers initially discussed the relevant mental health conditions, including the signs, symptoms, and duration of the mental health conditions, and then explained the instruments in a simplified manner. Moreover, during supervision sessions, if the peer responders shared any challenges of using these psychometric tools, those were resolved during those sessions.

Module 2 covered community-based inclusive MH support, aims and advantages of peer support, characteristics, and techniques of psycho-social support (active listening, empathy, non-judgmental approach, summarising, paraphrasing), referral, and follow-up. Module 3 encompassed the importance of MH awareness in eliminating stigma, techniques of self-care, and basic knowledge of MH from different perspectives, such as disability, gender-based violence and crisis.

#### Phase 2: Awareness and sensitisation to the community

Following training, peer responders went door-to-door in their communities to meet recipients of CDD services. These visits secured their acceptance in their catchment areas, which included urban slums, semi-urban slums, and rural areas. Peer responders arranged regular courtyard meetings with persons with disabilities and their caregivers to raise awareness of their role, MH and self-care. They also held regular meetings with community workers, teachers, religious leaders, and representatives of local government agencies.

#### Phase 3: Responding to primary mental health needs

After phases 1 and 2, peer responders made home visits to provide needs-based services and provided their contact numbers for round-the-clock tele-response. When peer responders found any sign of distress and low mood among the persons with disabilities in their communities, they administered screening tools such as DASS-21, PHQ-9, and GAD-7, which they were trained through the capacity building process. For mild to moderate cases, immediate psycho-social support was provided by peer responders. The peer responders provided periodic visits and gave their phone numbers to the service recipients so that they could access the peer responders’ support. In terms of severe cases, urgent referrals were made as soon as identified, and hotline numbers for professional help were given as well.

#### Phase 4: Ensuring better mental health services for needs and referrals

Dedicated hotline numbers were established with the help of CDD MH professionals to provide immediate and need-based tele-mental health support to persons with disabilities. Moreover, referrals were made to the nearest specialised hospitals if needed, with regular follow-ups by peer responders. MH camps were organised periodically with experts, increasing the acceptance and accessibility of the services to attend to the severe cases.

#### Phase 5: Supervision and refresher training

Peer responders received 2 hours of supervision each week, conducted by two psychologists who were trainers, to discuss critical cases. The supervision was provided in a group setting through online meetings conducted using Zoom video communication software. The intervention development team had a structured guideline for supervision; however, it was also improvised based on feedback from the peer responders. During these meetings, the peer responders shared their daily challenges and experiences of implementing training knowledge at the community level. The supervisors then offered guidance based on guidelines and real-life experiences. MH experts from the National Mental Health Institute (NIMH) and CDD delivered refresher training 5 months after the first training to strengthen the knowledge of peer responders’ understanding of MH issues. A monitoring committee regularly visited the catchment areas to observe the PRT programme activities and collect feedback from participants.

### Study population

The assessment of the feasibility of PRT, a multi-component intervention programme, was conducted using focus group discussions and key informant interviews among relevant stakeholders following Bowen et al.’s feasibility study framework [55, 56]. The indicators included in this study were acceptability, demand, implementation, and integration. Data was collected from peer responders and representatives of affiliated OPDs and DSOs. Furthermore, two interviews were conducted with peer responders who dropped out of the programme. The trainers (MH specialists) involved in developing and delivering the PRT modules were also interviewed. The study also involved CDD and CBM Global staff, who were instrumental in project delivery and promotion of disability care in Bangladesh. Study participants were selected purposively from the list held by CDD.

### Data collection

The data collection was conducted by the research team consisting of three team members with degrees in global mental health, public health and medicine with prior experience in leading qualitative research. In-depth key informant interviews (KIIs) and focus group discussions (FGDs) were used to collect data. Semi-structured interview guides were developed, comprised of questions related to study objectives (Appendix B). KII and FGDs were both employed while collecting data from peer responders. Trainers, representatives of OPDs and DSOs, and CDD and CBM officials participated in KIIs. Before the data collection, peer responders and representatives of OPDs and DSOs were approached through telephone calls detailing the study’s purpose. Other participants were approached through email with study-related information. All the participants were asked to share their interest in being involved in the study. Thus, the participants shared their preferred schedules with the research team through their respective communication pathways.

Representatives of the OPDs, DSOs, CDD and CBM were interviewed regarding perceptions of the PRT programme, its challenges, recommendations, and the potential to scale the programme nationwide. Researchers gathered peer responders’ overall experiences, including their perceptions of the PRT programme, experiences in providing MH support in the community, and perceived challenges to receiving training and providing services. They were also asked about opportunities and recommendations for scaling up the PRT programme for persons with disabilities in the future. Interviews were conducted in the local language (Bangla) by a research team experienced in qualitative data collection. The interviews lasted for 40–50 minutes, and FGDs lasted for 110–120 minutes on average and were audio recorded along with field notes. Face-to-face meetings were conducted among peer responders and representative OPDs and DSOs at their preferred places in Dhaka and Chattogram. Online Zoom meetings were conducted among trainers and CBM and CDD officials. Following each interview and discussion, the research team completed the transcription and coding simultaneously. Data collection ended when no new data emerged from interviews (data saturation). All the audio recordings were transcribed by the research team.

### Data analysis

Thematic analysis was applied using both inductive and deductive techniques. Three research team members involved in data collection and proficient both in English and Bengali independently and manually coded the transcripts, translating significant findings and quotes into English. The research team became familiar with transcripts through iterative reading. Initial coding categorised data points that corresponded with study objectives. Individual codes were summarised under themes and subthemes. Relevant quotes were translated from Bangla to English. Data triangulation was assured by meeting with supervisors, comparing the data to current research, and considering the perspectives of the various stakeholders in the study. The team regularly consulted with co-investigators and resolved discrepancies. This study adhered to the consolidated reporting criteria for qualitative studies (COREQ) (Appendix C) [57].

## Result

In total, 31 stakeholders participated in the study. Most stakeholders were peer responders (*n* = 18) from Dhaka and Chattogram. (Tables 1 and 2).

**Table 1:** Characteristics of peer responders/ participants (*n* = 18)

Identification and gender of Peer responders	Name of the organisations	Location	Number of participants	Type of disability	Qualitative method
PR01 (Female) PR02 (Female)	Disabled Child Foundation (DCF)	Khilgaon, Dhaka	2	Lower limb disability	KII
PR03 (Male) PR04 (Female)	Alor Chhaya Protibondhi Songstha	Mirpur, Dhaka	2	Lower limb disability	KII
PR05 (Female) PR06 (Male)	Jugantar Samaj Unnayan Sangstha (JSUS)	Banshkhali, Chattogram	2	Lower limb disability	FGD 1
PR07 (Female)	Jugantar Samaj Unnayan Sangstha (JSUS)	Banshkhali, Chattogram	1 (Dropout)	Lower limb disability	KII
PR08 (Female) PR09 (Male)	Association for Women Empowerment and Child Rights (AWAC)	Rangunia, Chattogram	2	Lower limb disability	FGD 1
PR10 (Male) PR11 (Female)	Alliance of Urban DPO’s in Chittagong (AUDC)	Pahartoly, Chattogram	2	Visual and Lower limb disability	FGD 1
PR12 (Male) PR13 (Male) PR14 (Male)	Disabled Development and Research Center (DDRC)	Lalkhanbazar, Chattogram	3	Visual and Lower limb disability	FGD 2
PR15 (Male) PR16 (Female) PR17 (Female)	Centre for Disables Concern (CDC)	Bagmonirum, Chattogram	3	Lower limb disability	FGD 2
PR18 (Male)	Centre for Disables Concern (CDC)	Bagmonirum,	1 (Dropout)	Lower limb disability	KII
	Total		18

**Table 2:** Characteristics of all other stakeholders (*n* = 13)

Identification of stakeholders	Name of the organisations	Location	Number of participants	Foci	Qualitative methods
Trainer 01 (Male) Trainer 02 (Male)	CDD	Dhaka	2	Trainer (Psychologists)	KII
OPD 01 (Male) OPD02 (Female)	Disabled Child Foundation (DCF)	Dhaka	2	Representative of Partner OPDs	KII
OPD 03 (Male) OPD 04 (Male)	Alor Chhaya Protibondhi Songstha	Dhaka	2	Representative of Partner OPDs	
DSO 01 (Male)	Centre for Disables Concern (CDC)	Chattogram	1	Representative of Partner DSOs	KII
DSO 02 (Male)	Alliance of Urban DPO’s in Chittagong (AUDC)	Chattogram	1	Representative of Partner DSOs	
DSO 03 (Female)	Disabled Development and Research Center (DDRC)	Chattogram	1	Representative of Partner DSOs	
CBM officials 01 (male) CBM official 02 (Female) CDD Official 01 (Female) CDD Official 02 (Male)	CBM Global CDD	Dhaka	4	Official of CBM and CDD	KII
	Total		**13**		

Three major themes (Table 3) emerged from analyses: (1) Acceptability and relevance of the PRT programme, (2) Perceived benefits of the programme, and (3) Challenges and barriers. Each theme is presented below along with representative quotes.^1^

**Table 3:** Themes and subthemes

Themes and subthemes	
1. Acceptability and relevance of the PRT programme	
a. Perspectives	
b. Motivation for participating	
c. Acceptability of programme activities	
2. Perceived benefits of the programme a. Increased MH literacy of persons with disabilities	
b. Accessible MH support in the community	
c. Positive impact on the well-being of the peer responders	
3. Challenges of implementing PRT programme	
a. Challenges of receiving PRT	
b. Challenges of providing PRT services	
c. Logistical Challenges	

### Theme 1: Acceptability and relevance of the PRT programme

#### Perspectives

All stakeholders generally shared positive perceptions of the PRT programme. Peer responders described an inclusive training programme that paved the way for learning about MH and the rights of persons with disabilities. Representatives of OPDs and DSOs perceived a learning environment as effective for peer responders’ capacity building. One peer responder said,

*“The training environment was welcoming and friendly. There were around 20 to 21 participants in the training. The understanding among participants was outstanding. We all shared a heartfelt bond and never felt monotonous at any point. " – PR01, age 24, Lower limb disability.*

Trainers, CDD and CBM officials, on the other hand, perceived that capacity-building opportunities such as the peer-responders training programme would help create an alternative workforce to address the mental health needs of persons with disabilities and develop an advanced referral system by ensuring inclusive and accessible mental health services in the community. Moreover, PRT would empower the persons with disabilities and ensure peer support as they had similar life experiences, which would eventually help them connect with the persons with disabilities spiritually. One of the trainers stated,

*“Our vision was to build the capacity of persons with disabilities for mental health and who would get back to their community to provide need-based services. Throughout the training, one would learn to identify mental health issues, provide referrals, and increase awareness about mental health, ultimately ensuring easy access to mental health services for persons with disabilities through their peers.”- Trainer 01, age 38, Psychologist.*

#### Motivation for participating

When asked about motivations for participating in the programme, most peer responders felt that understanding MH was essential for themselves and other people with disabilities. One peer responder stated,

*“The president of our OPD informed me about the training, and it intrigued me. While visiting CDD, I learned more about it and became determined to participate. I always felt that caring for my mental health was essential for my physical health. However, I had little idea about it. So, I participated in the training to better understand it.” – PR02, age 28, Lower limb disability.*

Another group was willing to join the training to challenge negative perceptions about MH and disability in the community. Some mentioned that most people in their community refer to persons with disabilities as “mad”, even if they are mentally well. They believed a deeper understanding of disability and MH conditions could help many people in their communities.

Trainers, CDD and CBM officials stated that peer responders were motivated to join the programme because it provided a capacity-building opportunity and a way to empower themselves. Trainers added that regular attendance at training and supervision sessions reflected the motivation of peer responders. Following sessions, they participated in the DSO’s activities with enthusiasm. One trainer said,

*“Peer responders were happy to be trained in mental health and their rights. They were very active during supervision sessions. They shared their positive experiences and challenges. Their attendance and interaction were satisfactory.” – Trainer 02, age 37, Psychologist.*

One CDD official added that peer responders who had experienced disability felt a strong need to understand well-being, and supporting another person with a disability encouraged their participation in the programme. Trainers also added that positive feedback from the service recipients increased motivation among peer responders.

*“According to my observations, peer responders liked the opportunity to work with persons with disabilities in the community. They knew the challenges a person has to experience when they have any kind of disability. The ability to support those people in their vulnerable conditions, hearing about their experiences and solving their psychological issues throughout the process motivated the peer responders to support the training activity. Moreover, they received positive feedback and blessings from the service recipients, which they shared with us during the supervision sessions.”- Trainer 01, age 38, Psychologist.*

#### Acceptability of programme activities

Peer responders perceived the training contents as relevant for learning the basics of MH conditions to support the community. Most peer responders mentioned learning about basic counselling techniques and managing common MH conditions through the PRT programme. One stated,

*“There are many mental health issues we were previously unaware of… We learned about the DASS-21, through which we can screen a person’s mental state. Also, we learned when to refer the client to a mental health specialist.” – PR01, age 24, Lower limb disability.*

Some peer responders felt the training contents reflected their psychological well-being, provided a sense of social protection, and provided insight into their fundamental rights as human beings. According to one peer responder,

*“We learned about different types of disabilities and our vulnerability to mental health problems. I learned about laws regarding disabilities. Before training, we did not know our rights. Now, we can raise our voices when we experience discrimination.” - PR04, age 30, Lower limb disability.*

Training facilitators made a similar observation regarding training content and process. Trainers said the sessions were interactive and peer responders enjoyed them, especially roleplays and group discussions. Peer responders provided positive feedback after training sessions and agreed that training sessions were lively and participatory*.*

Regarding service delivery, some peer responders insisted door-to-door community visits were crucial because these allowed people to share their inner thoughts and feel supported. Reportedly, courtyard meetings raised MH awareness and reduced community prejudices against people with disabilities. Community members who did not even speak with their families trusted and confided in empathic peer responders. Skills learned from the training session helped peer responders communicate correctly. One shared:

*“We learned about creating self-confidence and handling superstitions without hurting community people's beliefs. This initiative was beneficial as we could openly discuss mental health conditions and perceived superstitions with our target participants in a group setting.” – PR10,* age 27, *Lower limb disability.*

Peer responders also shared that awareness raising and door-to-door visits sensitised recipients to MH. Participants sought support for themselves, immediate family members, and relatives. Peer responders also appreciated their trainers’ efforts. Due to the trainers’ expertise, peer responders could complete their tasks. Trainees acknowledged the helpfulness of regular supervision sessions, as they could discuss challenges with trainer psychologists. Sharing field experiences with trainers created hands-on learning of the contents taught. A peer responder said,

*“Our trainers were excellent, conducting 3-4 supervision sessions a month. When we experienced any challenges, we discussed the issues at supervision sessions. When we could not understand cases, our supervisors discussed those issues in class so we all could understand.” – PR03, age 20, Lower limb disability.*

### Theme 2: Perceived benefits of the programme

#### Increased MH literacy of persons with disabilities

Peer responders reported the programme activities as helpful, especially for people with physical and sensory impairments. Their MH literacy increased as peer responders shared knowledge of MH and available MH services through flashcards, leaflets, courtyard meetings, etc. Peer responders believed that such approaches were instrumental in eradicating stigma while providing basic MH services. Peer responders reported that training activities influenced the target participants’ MH-seeking behaviour, and the community’s demand for MH-care echoes the statement’s relevance. One peer responder said,

*“Mental health was a new concept for people with disabilities and their families. After our meetings and door-to-door visits, people with disabilities constantly asked questions and shared their issues about themselves and their family members. They asked for referrals to psychologists and psychiatrists for advanced level services.” -PR04, age 30, Lower limb disability.*

CDD officials, representative OPDs, and trainers confirmed that peer responders increased awareness among persons with disabilities to identify their issues and seek care. A CDD official said there weren’t any MH awareness-raising activities in the community before. Therefore, peer responders played a positive role for the community, especially for those who used mobile phone services. Stakeholders also highlighted that, as PRT services were implemented during the COVID-19 pandemic, having mobile phones helped persons with disabilities access tele-mental health support from the CDD hotline numbers and peer responders easily. The inclusion of tele-mental health services helped overcome the geographical and other logistical barriers for a person with disabilities.

*“During the COVID-19 pandemic, peer responders played an incredible role in ensuring basic mental health services. Peer responders shared their phone numbers with the beneficiaries so that they could ask for help any time they wanted. Moreover, 24/7 tele-response services were provided through CDD hotline numbers by psychologists. Peer responders informed the persons with disabilities about the hotline number and the process of seeking services”- CDD official 02, Male, 10 years of work experience in the disability sector.*

Persons with disabilities are more vulnerable to developing severe MH issues due to difficulties sharing emotions and accessing MH services. Stakeholders postulated that peer responders could contribute to preventing severe conditions by identifying and managing early symptoms of MH conditions. They also believed peer responders played a significant role in alleviating inner distress.

#### Accessible MH support in the community

Some stakeholders asserted that MH services could be accessible in the community through peer responders. Peer responders provide needs-based support, such as primary counselling or referral, especially in rural areas where service is unavailable. An OPD representative said,

*“Peer responders have a vital role to play. Although mental health services are available in cities, they are insufficient in rural areas. District-level or tertiary hospitals are not available in remote areas. People cannot access those services due to distance and lack of other resources. Individuals with disabilities can, however, easily access mental health services from peer responders available in the community.” - OPD 02, Male, Lower limb disability.*

The training also helped peer responders to identify severe cases such as schizophrenia, major depressive disorder, and obsessive-compulsive disorder (OCD). Another peer responder stated,

*“We learned about OCD in our training. Before that, we did not know why somebody would be obsessed with cleaning, commonly known as “suchibayu” in our society. Now we know how to support that person instead of stigmatising them. We learned to talk to that person respectfully and handle the situation properly.” – PR08, age 27, Lower limb disability.*

Peer responders shared similar perceptions, mentioning that they were able to reach the community’s disadvantaged populations. They visited people living in slums, individuals with low education, and those with unaddressed issues to teach them about MH and help them manage some of their MH issues.

Poor access to mental health support was a major challenge regarding the mental healthcare-seeking behaviour of persons with disabilities. Stakeholders shared that the peer responder training model overcame geographical, logistical, and service accessibility challenges. The service recipients of peer responders did not only receive psycho-social support from peer responders but also obtained tele-mental health support from CDD and visited mental health camps to meet psychologists of CDD after getting referred by peer responders. Moreover, some of them were referred to the National Mental Health Institute (NIMH) or other tertiary care facilities as CDD has an existing collaboration with NIMH. One CDD official stated,

*“People with disabilities often face challenges in accessing health services, particularly mental health, due to various factors. They come from low-income families and have limited access to services, such as community clinics. Access to mental health services is limited, and many require accessible transport, devices, and caregivers. Parents often work full-time, making it difficult to dedicate time to their children. Public transportation is not always suitable for people with disabilities, and communication is a significant barrier. The lack of accessibility and high costs further complicate access to mental health services for people with disabilities. Therefore, peer responders played a significant role in reducing these challenges as they could easily reach the persons with disabilities through door-to-door visits, provided basic counselling to their mental health challenges and referred severe cases to the specialized facilities and professionals of mental health camps.” - CDD official 01, Female, 19 years of work experience in the disability sector.*

#### Positive impact on the well-being of the peer responders

Peer responders felt service activities were promising not only for community members but also for themselves. Engaging in programme activities improved the well-being of peer responders by increasing self-confidence and fulfilment through contributing to the community. One responder stated,

*“Most people with physical and sensory difficulties could not access mental health services before, as they didn’t know where these were available. But through our support, they learned what to do about their issues, which made us happy. Before participating in the training, I used to feel low about myself. But my job as a peer responder has provided me with the courage and confidence that I can also do something for others.” – PR03, age 20, Lower limb disability.*

Some peer responders perceived their role as spokespersons for their peers with disabilities as beneficial and important as they experienced societal discrimination. OPD and DSO representatives and facilitators agreed that training positively impacted peer responders as they became more conscious of their well-being. One DSO representative recalled a peer responder who struggled with suicidality before the training. They encouraged the individual to attend the training and engage in social work activities to benefit people with disabilities. Training increased their self-confidence, pride, and overall well-being, positively impacting their MH.

### Theme 3: Challenges of implementing PRT programme

#### Challenges of receiving PRT

The peer responders shared some challenges they experienced in the PRT programme. They stated that the training duration was inadequate to understand all the course materials. Some trainees added that no previous exposure to MH-related knowledge was a huge barrier to understanding the contents. According to a peer responder,

*“It would have been better if the training period had been longer because we had no previous academic knowledge about mental health. Five days were insufficient to comprehend everything, especially recognising mental health conditions. I was worried that if I did not memorise everything, I would not be able to deliver the best service.”- PR02, age 28, Lower limb disability.*

Representatives of partner OPDs and DSOs, and trainers made similar observations. They claimed that because peer responders had low socio-economic and educational statuses, training materials could sometimes be difficult to understand. Differences in recipients’ literacy levels further intensified challenges to full participation in the training. Some OPD representatives felt the training required more socio-cultural customisation. One stakeholder stated,

*“Some peer responders shared that they had trouble understanding training materials. Some content, in my opinion, was difficult to understand and implement and needed socio-cultural customisation. They should receive more lessons for conveying training knowledge in a local context because they would be interacting with members of their local communities.” -OPD 01, Female, Visual impairment.*

#### Challenges of providing PRT services

While providing MH services, peer responders experienced several challenges, such as pandemic-related concerns, poor understanding of MH in the community, acceptance, and logistical issues. As the PRT programme was initiated to provide support during the COVID-19 pandemic, all stakeholders, including peer responders, faced challenges in providing home visits during the lockdown. Because of social distancing protocols, it was hard to reach people with disabilities in person. One peer responder stated,

*“With multiple pandemic outbreaks ongoing, it was difficult to conduct door-to-door visits. Due to fear of infection, people did not want us to enter their homes. Some of them forced us to leave the residence even before we could speak to them.” – PR03, age 20, Lower limb disability.*

Trainees shared difficulties in accessing homes during the starting phase of the programme as they were perceived as outsiders by the community. CDD officials and trainers shared that it was also challenging for peer responders to ensure privacy while providing counselling services, as the service users were often surrounded by other family members.

*“We went to their houses every day, but people avoided us because they didn’t want to talk to us. They thought we were strangers and asked about our identities. They also wanted to contact our OPDs before allowing us into their houses.” - PR13, age 32, Lower limb disability.*

CDD officials added that peer responders with visual impairments felt significantly challenged in terms of applying the psychometric assessment questionnaire and demonstrating behaviour change communication materials, such as flashcards and leaflets. One peer responder dropped out due to mobility difficulties.

Most peer responders reported that providing MH services to the community was challenging due to the low MH literacy of people with disabilities and their family members. Due to the stigma surrounding MH issues, family members sometimes discouraged them from receiving mental health support, making it challenging to conduct community courtyard meetings and door-to-door visits. A peer responder said,

*“When we talked to someone suffering from a severe mental health condition, people used to say we were talking to a crazy person and that they would not benefit from our support. People in the community discouraged us from providing peer responder services.” -PR03, age 20, Lower limb disability.*

Other stakeholders reflected similar observations and stated that ensuring treatment adherence was a major challenge due to low MH literacy. Most people stopped taking medicines and did not attend follow-up consultations with psychiatrists in the medical college hospital. One peer responder stated,

*“We have seen people unwilling to visit hospitals even after getting referrals. Those who visited mental health specialists were not adhering to the prescriptions. Due to a lack of understanding, they stopped taking medicines after a few days. They did not even attend follow-up sessions.” – PR14, age 29, Lower limb disability.*

#### Logistical challenges

Some peer responders stated that being a person with a disability made providing services difficult. Long walks and a lack of convenient transportation options limited service provision. Peer responders faced greater challenges in community outreach services during outbreaks of natural disasters. One said,

*“We faced some challenges in transportation. We had to walk far for home visits, which was tricky with our crutches. It became more difficult during rainy seasons or stormy weather. Some of us were unable to use buses or rickshaws because getting on them is incredibly hard without assistance.” – PR15, age 38, Lower limb disability.*

Lack of financial support was another challenge identified. Most peer responders came from low-income families and thus did not get adequate financial support from their families. As the PRT programme provided no remuneration or financial support, it affected the continuation of service provisions except for transport costs and phone bills, it affected the continuation of service provisions. Due to their physical limitations and lack of financial support for transportation, they could not sustain peer responder services. A peer responder said,

*“For the benefit of others, we voluntarily offered peer responder services without any financial assistance. My husband is unemployed, and I have two children with disabilities. My financial situation is not great. So, I could not offer services in far-off places that would be financially challenging. Hence, continuing the services without any financial assistance would be very difficult.” - PR01, age 24, Lower limb disability.*

National and international CBM Global and CDD stakeholders indicated that sustaining MH services by peer responders would be challenging due to a lack of financial support. Because of the project-based nature of the programme, it was impossible to provide proper remuneration for peer responders who worked voluntarily. This PRT programme would be challenging to sustain economically in the long run. A CBM official said,

*“PRT programme was a time-sensitive programme. We acknowledged that they faced financial challenges in carrying out training activities. Most of the peer responders are from low-income families, so it becomes difficult for them to manage the transportation and communication expenses needed to deliver the service. Therefore, we worked with those people with disabilities who wanted to work voluntarily as peer responders. However, in the long run, we must ensure some financial mechanism to sustain the services provided by the peer responders.” – CBM official 01, Male, 15 years of experience in the disability sector.*

People with disabilities in the community also asked for financial support from the peer responders. According to a peer responder,

*“During our visits, people with disabilities in the community also asked for money, being financially vulnerable. We had to convince them that talking to us would benefit their health. When we tried to convince them with information about the importance of taking care of mental health, some of them agreed and stopped asking for money. But some people stopped taking our service midway.” - PR 11, age 28, Visual impairment.*

The findings also included a pragmatic implementation plan with recommendations to strengthen the programme (Table 4).

**Table 4:** The programmatic implications of the findings in the context of Bangladesh

Areas of interest	Constraints/Barriers (challenges)	Existing facilitators (benefits)	Opportunities for interventions
a. Training programme content	• Inadequate/ short training duration • Lack of previous exposure to MH-related knowledge • Difficulty in understanding training materials/ contents • The training was not socio-culturally customised	• MH literacy increased • Social stigma related to MH reduced • Improvement of peer responders’ well-being • Participatory nature of the PRT programmes, such as-group activities, roleplays and Q&A sessions • Strengthening peer responders’ professional capabilities	• Increasing the number and duration of the training sessions • Including more content • Practical activities/fieldwork to reduce monotony
b. Service delivery	• Poor understanding and acceptance of MH in the community • Challenges in providing home visits during COVID-19 related lockdown • Peer responders were perceived as outsiders by the community • Difficulty in ensuring privacy while providing counselling services • Difficulty in applying psychometric assessment tools and demonstrating behavioural change communication materials for peer responders with visual impairments • Difficulties in movement from one place to another • Social stigma related to MH and disabilities • Non-compliance/ non-adherence to follow-up consultations • Lack/high cost of transportation	• Contribution to preventing severe MH issues • Identifying and managing mild MH issues • Necessary referral services • Sense of contributing to the community • Positive influence on MH-seeking behaviour • Increased awareness about MH	• Financial support • Capacity building of the peer responders for future peer support programmes • Formal recognition and provision of ID cards • Community awareness programmes
c. Integration and expansion	• Lack of convenient transportation services for the peer responders • Lack of financial support • Difficulties in providing remuneration to the peer responders due to the small-scale project-based nature of the PRT programme	• Applicable for the disadvantaged/marginalised group of people • Basic understanding of MH issues and referral among peer responders • Available telemedicine and tele-counselling services • Community sensitisation • Medical camps	• Integration of PRT services in mainstream/ existing health system • Multi-sectoral collaboration with governmental and NGOs • Expanding accessibility and availability of psychiatric medications • Large-scale training programme to build the capacity of peer responders • Further research on the effectiveness of the PRT programme • Increasing awareness about the PRT programme at the national level

## Discussion

Peer support is a well-recognised MH concept supported by international evidence, especially from LMICs [58, 59]. A peer is a person who shares comparable characteristics in terms of social status or lived experiences and is highly empathic with a capacity for reassurance and support that a person with shared experiences can offer to one another in a reciprocal relationship [34, 60].

The prospect of peer support in healthcare decision-making and communication was highlighted in the UNCRPD. However, limited research in Bangladesh has highlighted the availability and viability of peer-led MH programmes for this group [61–63]. The stakeholders in this study highlighted a wide range of perspectives and understanding from their point of view. The peer responders added their concerns regarding the implementation of training at the field level, and representatives of OPDs and DSOs shared their perspectives as they were more knowledgeable about their community. On the other hand, trainers and officials of CDD and CBM added their concerns from administrative and policy perspectives. This study adds to the literature about the benefits and challenges of peer support programmes and services and provides recommendations for scaling-up such programmes in LMICs like Bangladesh.

Our findings suggest the PRT programme was acceptable to all stakeholders. Stakeholders perceived the PRT programme as an applicable way of providing MH services to the marginalised population. They felt confident that this non-specialist workforce could build capacity for MH with appropriate funding and support. MH-related training for non-specialists (such as community health workers, lay health counsellors, persons with lived experience of MH issues, and caregivers of children with neurodevelopmental disorders) is acceptable in resource-poor settings like Ethiopia, India, Nepal, South Africa, and Uganda [64–68]. Previously, in Bangladesh, caregiving and skill-building interventions integrated with psycho-social services for mothers of children with autism reduced MH symptoms and improved quality of life [67]. Our findings indicate that training on identification, basic psycho-social support, and referral techniques combined with activities such as door-to-door visits was appropriate for delivering basic MH services in communities with people with disabilities. A study conducted in India similarly successfully trained an MH workforce to conduct door-to-door visits and provide counselling or referrals [69]. Our study was able to further these findings by focusing on promoting people with disabilities as peer responders. Our findings show that roleplays, group discussions and participatory question-answer sessions were enjoyable and effective. A systematic review of WHO guidance on MH training found that interactive group learning was one of the most conventional methods for ensuring low dropout rates [70]. Previous reviews also suggest that community-based awareness programmes delivered by non-specialists have the potential to reduce MH stigma, which aligns with our study findings [71].

Our findings show that peer responders were highly motivated to join the training as they viewed it as an opportunity to support their peers. Trainees believed this programme would strengthen their professional capabilities and improve their well-being. Other studies have also concluded that high motivation and enthusiasm are crucial for the proper implementation and sustainability of any MH intervention delivered by non-specialists [72, 73]. Findings highlighted the multi-dimensional usefulness of peer support activities, which included increasing MH literacy, MH-seeking behaviour, and the accessibility of MH services. All stakeholders reported that training activities increased the confidence of peer responders and contributed to reducing stigma among peer responders and recipients. In India, grassroots-level programmes involving non-specialists reduced depressive symptoms and improved MH literacy and care-seeking in rural communities [74]. A multi-country study conducted in Ethiopia, India, Nepal, South Africa, and Uganda reported that task sharing by involving non-specialists could effectively increase the affordability and accessibility of MH services [64]. Through sharing experiences with clients, a non-specialist peer support worker can develop a positive outlook on life and improve self-esteem [75, 76]. Some studies show that non-specialist MH-care providers have become the first line of support in reducing the MH treatment gap in poor resource settings [64, 73, 74]. However, to ensure safeguarding against low-quality service provision and “task dumping,” which can be characterized by imposing additional workload on non-specialist service providers, there must be a well-trained supervisory team available to regulate mental health care in those areas. We plan to address this issue in future studies where a similar model will be implemented.

Inadequate training duration, low educational status, and lack of previous exposure to MH knowledge were challenges indicated by stakeholders in implementing PRT programme activities. The acquisition of knowledge and skills varies based on literacy, capacity for understanding, motivation, and the learning environment. Robust information delivery and adequate training durations are crucial for success [77]. WHO caregiver skill training programmes in rural Ethiopia also reported that discrepancies in educational status impacted the attainment of training skills and knowledge [68]. Educational backgrounds should be assessed before training, and trainees should be grouped by knowledge level. Supervision and refresher training can also contribute to knowledge retention [78].

Lack of knowledge about non-specialist MH-care providers and treatment, stigma, and low MH literacy in the community lead to the underutilisation of MH services [79, 80]. This may be due to the lack of MH-related resources and community awareness programmes [80]. Increased government support for public awareness campaigns and services at the primary healthcare level is crucial [81–83].

Geographical distance, lack of convenient transport, and absence of financial support were major barriers for peer responders. Unfortunately, most LMICs fail to ensure basic assistance for people with disabilities, limiting their mobility and access to services [81–84]. Additionally, a lack of opportunity to engage in the economic sector creates financial strain. A study from Tanzania showed families and communities monetarily supplemented the voluntary engagements of non-specialist health workers [72, 85]. As low financial support negatively affects the providers’ motivation, it is essential to encourage them through professional development opportunities, monetary support, and non-monetary remuneration, such as transportation and other logistical supplies. These can reinforce existing altruism, amplify commitment, and improve the sustainability of non-specialist peer support providers. Moreover, counting on the findings of this study, policymakers and relevant stakeholders can plan future intervention strategies by creating job opportunities with proper remuneration, which would pave a path forward to create an alternative workforce formed with trained persons with disabilities for delivering mental health services not only to their peers but also to their whole community.

To improve the PRT programme, stakeholders stressed the need to increase the duration of the training and include more practical activities, more content on communication skills and disability rights, and guidelines for proper referrals. Community-level health workers who received an MH training programme in Nepal expressed similar wishes [77]. Improving the communication skills of MH service providers is crucial for client satisfaction, alleviating distress, increasing therapeutic adherence, and improving MH outcomes [86]. Programme facilitators should focus on supervision, monitoring, eliciting feedback, and refresher training.

Building capacity through train-the-trainer models and formally recognising peer responders as MH advocates through community awareness activities was also recommended. Previous literature also emphasises the importance of advancing the capacity building of non-specialist care providers [87, 88]. A review noted that skill-building training should include assessment, communication skills, problem-solving, professional responsibilities and boundaries, and stress and emotion management strategies [87]. It is important to have different types of assistive support available, such as braille translations or hearing devices, for those with audio/visual impairments. The potential for formal recognition of community-level healthcare providers to gain trust and credibility at the community level and increase confidence was also discussed in the existing literature [88].

Stakeholders emphasised the expansion of the PRT programme through increased advocacy among policymakers and multi-sectoral collaboration. Increasing the availability of psychiatric medications and conducting large-scale training to increase the peer responder workforce were also suggested. Many countries have relied on people without MH training to deliver effective psychological interventions [34, 89]. Government-level advocacy is also important to increase funding for the sustainability of community-based MH services [90]. Additionally, increasing peer supporters in the community through internet-based training modalities and subsequent telehealth service provision can be adopted [30]. Integrating digital technology into training delivery and MH-care provision can increase the effective use of human resources and reduce costs [66, 91–93].

### Strengths and limitations

Some limitations of this study should be noted. The non-experimental design and small sample size limit the generalizability of findings. Purposive selection of participants potentially increased sampling bias by limiting recruitment reach. Participants selected for the PRT programme had 10 years of schooling on average, making it unclear if the intervention would be acceptable to less educated implementers, which is more typical of those living in rural and semi-urban areas. Recall bias is inherent to studies involving retrospective reporting of experiences in MH services. The study is not immune to social desirability bias, as the peer responders were not anonymous and were willing to continue their services through the PRT programme as it was perceived as a positive support for some of them. These could influence the participants to share positive feelings about the programme. Furthermore, peer responders were connected with the programme-implementing organisation and might fear that their negative response might affect their future training possibilities and other kinds of support. Due to the forward and back translation of interview guidelines and quotes from Bangla to English, some semantics may be lost.

Despite these limitations, our study has some notable strengths. This study is the first of its kind to generate evidence on the feasibility of delivering MH interventions by persons with disabilities, highlighting the potential of involving marginal populations in the provision of MH services. Qualitative interviews were conducted by third-party researchers not involved in developing and implementing the PRT programme. Qualitative inquiry enables deeper insights into programme benefits and challenges, which can assist in future scale-up.

## Conclusion

This study demonstrates that the PRT programme was an acceptable and feasible intervention for MH support to be delivered by people with disabilities. In a poor resource setting like Bangladesh, people with disabilities are extremely vulnerable to MH conditions, but access to MH services is incredibly low. Findings suggest that peer support has the potential to increase MH awareness in the community and may facilitate increased access to basic MH services. This programme also demonstrates the involvement of people with disabilities in meaningful social participation and their own rehabilitation and positive well-being.

### Supplementary Information

**Supplementary Material 1.**

## Data Availability

The datasets used and/or analysed during the current study are available from the corresponding author on reasonable request.

## Footnotes

1 Peer responders who participated in IDIs are denoted by “PR ##, age, type of disability” Trainers are referred to as “Trainer ##, age, profession.” Representative OPDs and DSOs are OPD ##, sex, type of disability and DSO ##, sex, type of disability. CDD and CBM officials are identified as “CBM Official ##, sex, years of experiences in disability sector” and “CDD Official ##, sex,  years of experiences in disability sector”.

## References

[1] Hossain MD (2014). Mental disorders in Bangladesh: a systematic review. BMC Psych.

[2] Nuri NN (2018). Pathways to care of patients with mental health problems in Bangladesh. Int J Ment Heal Syst.

[3] Hasan MT (2021). The current state of mental healthcare in Bangladesh: part 1–an updated country profile. BJPsych Int.

[4] Honey A, Emerson E, Llewellyn G (2011). The mental health of young people with disabilities: impact of social conditions. Soc Psychiatry Psychiatr Epidemiol.

[5] Milner A (2014). Employment status and mental health among persons with and without a disability: evidence from an Australian cohort study. J Epidemiol Community Health.

[6] Kavanagh AM (2016). Inequalities in socio-economic characteristics and health and wellbeing of men with and without disabilities: a cross-sectional analysis of the baseline wave of the Australian longitudinal study on male health. BMC Public Health.

[7] Emerson E (2021). Loneliness, social support, social isolation and wellbeing among working age adults with and without disability: cross-sectional study. Disabil Health J.

[8] Thompson S (2020). Disability inclusive development situational analysis for Bangladesh.

[9] Li CM (2014). Hearing impairment associated with depression in US adults, National Health and nutrition examination survey 2005-2010. JAMA Otolaryngol Head Neck Surg.

[10] Zhong B-L, Xu Y-M, Li Y (2022). Prevalence and unmet need for mental healthcare of major depressive disorder in community-dwelling Chinese people living with vision disability. Front Public Health.

[11] Sahu A (2016). Psychological effects of amputation: a review of studies from India. Ind Psychiatry J.

[12] Munaw MB, Tegegn MT (2022). Visual impairment and psychological distress among adults attending the University of Gondar tertiary eye care and training center, Northwest Ethiopia: a comparative cross-sectional study. PLoS One.

[13] van der Aa HP (2015). Major depressive and anxiety disorders in visually impaired older adults. Invest Ophthalmol Vis Sci.

[14] Fellinger J (2005). Mental distress and quality of life in a deaf population. Soc Psychiatry Psychiatr Epidemiol.

[15] Bruffaerts R (2012). Role of common mental and physical disorders in partial disability around the world. Br J Psychiatry.

[16] Court H (2014). Visual impairment is associated with physical and mental comorbidities in older adults: a cross-sectional study. BMC Med.

[17] Bonsaksen T, Brunes A, Heir T (2022). Post-traumatic stress disorder in people with visual impairment compared with the general population. Int J Environ Res Public Health.

[18] Jiang F (2020). The relationship between mental health conditions and hearing loss in low- and middle-income countries. Trop Med Int Health.

[19] Kuvalekar K (2015). Quality of life among persons with physical disability in Udupi taluk: a cross sectional study. J Family Med Prim Care.

[20] Bell D (2005). Well-being and quality of life: measuring the benefits of culture and sport: a literature review and thinkpiece. Scotish Executive Social Research.

[21] Brown R, Brown P, Bayer M (1994). A quality of life model: new challenges resulting from a six year study. Quality of life for persons with disabilities.

[22] Viemerö V, Krause C (1998). Quality of life in individuals with physical disabilities. Psychother Psychosom.

[23] Dalise S (2021). Psycho-social impact of social distancing and isolation due to the COVID-19 containment measures on patients with physical disabilities. Eur J Phys Rehabil Med.

[24] Kavanagh A (2022). Health and healthcare for people with disabilities in the UK during the COVID-19 pandemic. Disabil Health J.

[25] Ciciurkaite G, Marquez-Velarde G, Brown RL (2022). Stressors associated with the COVID-19 pandemic, disability, and mental health: considerations from the intermountain west. Stress Health.

[26] World Health Organization (2015). WHO global disability action plan 2014–2021: Better health for all people with disability.

[27] Baart J, Taaka F (2017). Barriers to healthcare services for people with disabilities in developing countries: a literature review. DCID.

[28] WHO (2016). Community health workers: a strategy to ensure access to primary health care services.

[29] Bruckner TA (2011). The mental health workforce gap in low- and middle-income countries: a needs-based approach. Bull World Health Organ.

[30] Gyee KM (2022). Empowering health workers and leveraging digital technology to improve access to mental health and epilepsy care: a longitudinal quasi-experimental study in Hlaing Thar Yar township. The lancet regional health - Southeast Asia.

[31] Singh OP (2018). Closing treatment gap of mental disorders in India: opportunity in new competency-based medical Council of India curriculum. Indian J Psychiatry.

[32] Sikander S (2020). Pakistan. Lancet Psych.

[33] Bunn M (2021). Supporting and sustaining nonspecialists to deliver mental health interventions in low- and middle-income countries: an umbrella review. Intervention.

[34] Shalaby RAH, Agyapong VIO (2020). Peer support in mental health: literature review. JMIR Ment Health.

[35] Kaplan K (2011). Internet peer support for individuals with psychiatric disabilities: a randomized controlled trial. Soc Sci Med.

[36] Nazari L, et al. Investigating the mediatory role of self-efficacy beliefs in the relationship between self-perception, peer support, and subjective well-being in visually impaired teenagers. Int J Health Sci. 2020;6(4)

[37] Kef S, Deković M (2004). The role of parental and peer support in adolescents well-being: a comparison of adolescents with and without a visual impairment. J Adolesc.

[38] Rotheram-Borus MJ (2015). Alcohol use, partner violence, and depression: a cluster randomized controlled trial among urban south African mothers over 3 years. Am J Prev Med.

[39] Bolton P (2003). Group interpersonal psychotherapy for depression in rural Uganda: a randomized controlled trial. JAMA.

[40] Chinman M (2015). A cluster randomized trial of adding peer specialists to intensive case management teams in the veterans health administration. J Behav Health Serv Res.

[41] Maddox S (2022). Effects of mental health training on capacity, willingness and engagement in peer-to-peer support in rural New South Wales. Health Promot J Austr.

[42] Reif S (2014). Peer recovery support for individuals with substance use disorders: assessing the evidence. Psychiatr Serv.

[43] Chapin RK (2013). Reclaiming joy: pilot evaluation of a mental health peer support program for older adults who receive Medicaid. Gerontologist.

[44] Byrom N (2018). An evaluation of a peer support intervention for student mental health. J Ment Health.

[45] Fortuna KL (2018). Feasibility, acceptability, and preliminary effectiveness of a peer-delivered and technology supported self-management intervention for older adults with serious mental illness. Psychiatry Q.

[46] Whiteman KL (2016). Systematic review of integrated general medical and psychiatric self-management interventions for adults with serious mental illness. Psychiatr Serv.

[47] Sokol R, Fisher E (2016). Peer support for the hardly reached: a systematic review. Am J Public Health.

[48] CDD (2022). Protecting and Empowering Persons with Disabilities in the Context of COVID-19 Pandemic.

[49] Kim H, Sefcik JS, Bradway C (2017). Characteristics of qualitative descriptive studies: a systematic review. Res Nurs Health.

[50] NDA (2009). Ethical guidance for research with people with disabilities.

[51] Durham J, Brolan CE, Mukandi B (2014). The convention on the rights of persons with disabilities: a foundation for ethical disability and health research in developing countries. Am J Public Health.

[52] CBM (2022). Bangladesh.

[53] Koly K (2022). Mental health and community-based rehabilitation: a qualitative description of the experiences and perspectives of service users and Carers in Bangladesh. Community Ment Health J.

[54] Mead S, MacNeil C (2006). Peer support: what makes it unique. Int J Psychosoc Rehabil.

[55] Bowen DJ (2009). How we design feasibility studies. Am J Prev Med.

[56] Naheed A (2017). Implementing a mental health care program and home-based training for mothers of children with autism spectrum disorder in an urban population in Bangladesh: protocol for a feasibility assessment study. JMIR Res Protoc.

[57] Tong A, Sainsbury P, Craig J (2007). Consolidated criteria for reporting qualitative research (COREQ): a 32-item checklist for interviews and focus groups. Int J Qual Health Care.

[58] Mahlke C (2014). Peer support in mental health services. Curr Opin Psychiatry.

[59] Repper J, Carter T (2011). A review of the literature on peer support in mental health services. J Ment Health.

[60] Penney D (2018). Defining “peer support”: implications for policy, practice, and research*.* Advocates for Human Potential.

[61] Pathare S, Shields LS (2012). Supported decision-making for persons with mental illness: a review. Public Health Rev.

[62] Mahomed F, Stein MA, Patel V (2018). Involuntary mental health treatment in the era of the United Nations convention on the rights of persons with disabilities. PLoS Med.

[63] Morrissey F (2012). The United Nations convention on the rights of persons with disabilities: a new approach to decision-making in mental health law. Eur J Health Law.

[64] Mendenhall E (2014). Acceptability and feasibility of using non-specialist health workers to deliver mental health care: stakeholder perceptions from the PRIME district sites in Ethiopia, India, Nepal, South Africa, and Uganda. Soc Sci Med.

[65] Sangraula M (2020). Feasibility of group problem management plus (PM+) to improve mental health and functioning of adults in earthquake-affected communities in Nepal. Epidemiol Psychiatr Sci.

[66] Muke SS (2019). Acceptability and feasibility of digital technology for training community health workers to deliver brief psychological treatment for depression in rural India. Asian J Psychiatr.

[67] Naheed A (2022). Feasibility of a school-based mental health program implementation to improve the status of depression and quality of life of mothers of children with autism spectrum disorders in urban Bangladesh: MENTHOL study. Global Mental Health.

[68] Tekola B (2020). Adapting and pre-testing the World Health Organization's caregiver skills training programme for autism and other developmental disorders in a very low-resource setting: findings from Ethiopia. Autism.

[69] Armstrong G (2011). A mental health training program for community health workers in India: impact on knowledge and attitudes. Int J Ment Heal Syst.

[70] Caulfield A (2019). WHO guidance on mental health training: a systematic review of the progress for non-specialist health workers. BMJ Open.

[71] Barnett ML (2018). Mobilizing community health workers to address mental health disparities for underserved populations: a systematic review. Admin Pol Ment Health.

[72] Greenspan JA (2013). Sources of community health worker motivation: a qualitative study in Morogoro region, Tanzania. Hum Resour Health.

[73] Liana L, Windarwati HD (2021). The effectivity role of community mental health worker for rehabilitation of mental health illness: a systematic review. Int J Health Sci.

[74] Shidhaye R (2017). The effect of VISHRAM, a grass-roots community-based mental health programme, on the treatment gap for depression in rural communities in India: a population-based study. Lancet Psychiatry.

[75] Beales A, Wilson J (2015). Peer support – the what, why, who, how and now. J Ment Health Train Educ Pract.

[76] MacLellan J (2015). Peer support Workers in Health: a qualitative Metasynthesis of their experiences. PLoS One.

[77] Angdembe M (2017). Situational analysis to inform development of primary care and community-based mental health services for severe mental disorders in Nepal. Int J Ment Heal Syst.

[78] Endale T (2020). Barriers and drivers to capacity-building in global mental health projects. Int J Ment Heal Syst.

[79] Jordans MJD (2016). Development and pilot testing of a mental healthcare plan in Nepal. Br J Psychiatry.

[80] Devkota G (2021). Factors affecting utilization of mental health services from primary health care (PHC) facilities of western hilly district of Nepal. PLoS One.

[81] Vergunst R (2017). Access to health care for persons with disabilities in rural South Africa. BMC Health Serv Res.

[82] Maart S, Jelsma J (2014). Disability and access to health care - a community based descriptive study. Disabil Rehabil.

[83] Kibria G (2020). Barriers to healthcare services for persons with disabilities in Bangladesh amid the COVID-19 pandemic. Public Health Pract (Oxf).

[84] World Health Organization (2021). Disability and health.

[85] Prytherch H (2013). Motivation and incentives of rural maternal and neonatal health care providers: a comparison of qualitative findings from Burkina Faso, Ghana and Tanzania. BMC Health Serv Res.

[86] Papageorgiou A, Loke YK, Fromage M (2017). Communication skills training for mental health professionals working with people with severe mental illness. Cochrane Database Syst Rev.

[87] Mistry SK (2021). Community health workers can provide psycho-social support to the people during COVID-19 and beyond in low- and middle- income countries. Front Public Health.

[88] Kissinger A (2022). Don’t change who we are but give us a chance: confronting the potential of community health worker certification for workforce recognition and exclusion. Arch Public Health.

[89] Koly KN (2021). Educational and training interventions aimed at healthcare Workers in the Detection and Management of people with mental health conditions in south and South-East Asia: a systematic review. Front Psychiatry.

[90] Castillo EG (2019). Community interventions to promote mental health and social equity. Curr Psychiatry Rep.

[91] Koly KN (2022). Exploring the potential of delivering mental health care services using digital technologies in Bangladesh: a qualitative analysis. Internet Interv.

[92] Bucci S, Schwannauer M, Berry N (2019). The digital revolution and its impact on mental health care. Psychol Psychother Theory Res Pract.

[93] Berry N, Lobban F, Bucci S (2019). A qualitative exploration of service user views about using digital health interventions for self-management in severe mental health problems. BMC Psychiatry.

