# Supplementary material for: Assessment of the feasibility of a community-based mental health training programme for persons with disabilities by non-specialists from different stakeholders’ perspectives in Bangladesh
Source: BMC Health Serv Res. 2024 Mar 4;24:270. doi: 10.1186/s12913-024-10742-5 (PMC10910748; doi:10.1186/s12913-024-10742-5)
Supplement: Supplementary file 1 — Supplementary Material 1. [file 12913_2024_10742_MOESM1_ESM.docx]

**Assessment of the feasibility of a community-based mental health training programme for persons with disabilities by non-specialists from different stakeholders’ perspectives in Bangladesh**

**Author (s): Kamrun Nahar Koly^1^, Jobaida Saba^1^, Enryka Christopher ^2^, Anan Nisat Nabela Hossain^1^, Taslima Akter^3^, Zakia Rahman^4^, Helal Uddin Ahmed^5^, Julian Eaton^6^**

1 Health System and Population Studies Division, International Centre for Diarrhoeal Disease Research, Bangladesh (icddr,b), Mohakhali, Dhaka 1212, Bangladesh

2 Trauma and Community Resilience Center, Boston Children's Hospital & Harvard Medical School, Boston, Massachusetts, United States of America

3 Centre for Disability in Development, Dhaka, Bangladesh

4 CBM Bangladesh Country Office (CBMBCO), Dhaka, Bangladesh

5 National Institute of Mental Health, Dhaka, Bangladesh

6 Centre for Global Mental Health, London School of Hygiene & Tropical Medicine, CBM Global, United Kingdom

United Kingdom

Email of the authors: Kamrun Nahar Koly <koly@icddrb.org>; Jobaida Saba <kazi.jobaida.saba@gmail.com>, Enryka Christopher <enrykachristopher@gmail.com>, Anan Nisat Nabela Hossain <ananhossain22@gmail.com>, Taslima Akter <[keya.cdd@gmail.com](mailto:keya.cdd@gmail.com)>, Zakia Rahman < [zakia.Rahman@cbm.org](mailto:zakia.Rahman@cbm.org)>, Helal Uddin Ahmed [soton73@gmail.com](mailto:soton73@gmail.com), Julian Eaton <Julian.Eaton@cbm-global.org>.

***Corresponding author**

Dr. Kamrun Nahar Koly

Associate Scientist

Health System and Population Studies Division, International Centre for Diarrhoeal Disease Research, Bangladesh (icddr, b), Mohakhali, Dhaka-1212, Bangladesh, Mobile: +8801717190955

E-mail: koly@icddrb.org, ORCiD ID: https://orcid.org/0000-0003-2466-8139.

**Appendix: 1.A Contents of PRT programme**

| Number and Name of the module | Components included | Topic covered | Process of delivery |
| --- | --- | --- | --- |
| Module 1:  Day 1:  1. Understanding of Basic Mental Health | - PowerPoint slides - Whiteboard - Flip chart - Supporting document related to the topic | - Introduction to mental health - Components of mental health - Mental health issues and symptoms of mental health issues | - Lecture - Open discussion - Group discussion |
| Module: 2  Day 2  Community-based mental health services | - Whiteboard - Poster paper - Flip chart - PowerPoint slides - Supporting document related to the topic | - Community-based mental health services - Aims and characteristics of peer responders - Ethics of peer responders - Psycho-social support and its techniques - Active listening | - Lecture - Open discussion - Group discussion - Question-answer session - Roleplay |
| Day 3  Community-based mental health services | - Whiteboard - Poster paper - Flip chart - PowerPoint slides - Supporting documents related to the topic | - Psycho-social support and its technique - Empathy - Non-judgmental approach - Summarising. - Paraphrasing | - Lecture - Open discussion - Group discussion - Question-answer session - Roleplay |
| Day 4  Community-based mental health services | - Videos - PowerPoint slides - Supporting document related to the topic | - Psycho-social support and management - Management of mental health conditions - Anxiety - Depression - Psychosis - Autism and autism-related issues - Psycho-social support in families - Process of follow-up and referrals - Increasing awareness on mental health - Courtyard meetings | - Lecture - Open discussion - Group discussion - Roleplay |
| Module 3  Day 5  Mental Health and social context | - Videos - PowerPoint slides - Flip chart - Supporting document related to the topic | - Community-level stigma and mental health - Mental health and disability - Mental health and gender-based violence - Mental health during crises - Self-care | - Lecture - Group discussion - Open discussion - Quiz - Presentation - Roleplay |

**Appendix 1.B: Semi structured interview guideline**

**Feasibility assessment of community-based inclusive mental health training programme in Bangladesh.**

Age

Gender

Area

1. Where did you first learn about this training? What made you decide to participate in the training?

2. Can you tell us what you learned from the peer responder training?

3. Do you apply what you have learned from the peer responder training? How?

4. Were there any challenges you experienced while taking the training? - If yes, please explain.

5. What training activity did you like the most? Why?

6. What training activity did you like the least? Why?

7. Were the peer responder training contents easy to understand?  If not, which content of the training module was difficult to understand or remember? Please explain what you did then.

8. If we arrange further training on mental health and mental healthcare, will you participate in the training? Why?

9. What kind of services do you provide as a peer responder? Where and to whom did you apply your knowledge and experience from the training? Can you please explain?

10. What are the challenges you experienced while serving as a peer responder?

11. Are the people interested in taking the services from you? What is their response to the services provided by you?

12. Do you think the peer responder training programme met its objectives well in training people to support the mental health of persons with disabilities?

- If yes, please explain.
- If no, please explain.

13. What can be done to make this peer responders training programme more inclusive for persons with disabilities? Or can you share your thoughts about how community-based inclusive mental health programmes can be scaled up throughout the country?

- How can it be beneficial for persons with disabilities?

14. Do you think we should add more information regarding mental health and disabilities in the training programme? / What can be done to improve the peer responders training (community-based inclusive mental health support programme)?

- What else do you want to know/ learn from this training program?
- What else should we include in the training for the new participants so that they can learn it properly?
- What is your recommendation regarding this?

15. Do you think this type of training should be given to all OPDs?

• If yes, please explain.

• If no, please explain.

**Appendix C: COREQ (Consolidated Criteria for Reporting Qualitative Research) Checklist**

| Topic | Item No. | Guide Questions/Description | Remark | Reported on Page No. |
| --- | --- | --- | --- | --- |
| Domain 1: Research team and reflexivity | | | | |
| Personal characteristics | | | | |
| Interviewer/facilitator | 1 | Which author/s conducted the interview or focus group? | Three team members (KNK, AH, and JS). | Methods – 5 |
| Credentials | 2 | What were the researcher’s credentials? E.g. PhD, MD | Mentioned at the end of the paper | Page 12 |
| Occupation | 3 | What was their occupation at the time of the study? | Mentioned at the end of the paper | Page 12 |
| Gender | 4 | Was the researcher male or female? | Female: 6; Male: 2 | N/A |
| Experience and training | 5 | What experience or training did the researcher have? | Lead author and other co-authors are experienced in conducting both qualitative and quantitative research designs. | N/A |
| Relationship with participants | | | | |
| Relationship established | 6 | Was a relationship established prior to study commencement? | No relationship with the participants was established before the commencement of the study. | N/A |
| Participant knowledge of the interviewer | 7 | What did the participants know about the researcher? e.g. personal goals, reasons for doing the research | KNK, JS, and AH introduced themselves to participants stating they were public health professionals and mental health researchers, etc. as well as describing the research team, the purpose of the project and answering any questions participants may have had about the study and those involved in it. | Method- 5 |
| Interviewer characteristics | 8 | What characteristics were reported about the interviewer/facilitator? e.g. Bias, assumptions, reasons and interests  in the research topic | The interviewers were public health professionals involved in multiple mental health research in Bangladesh and also had read the literature on peer lead mental health programmes from different countries . | Method 6 |
| Domain 2: Study design | | | | |
| Theoretical framework | | | | |
| Methodological orientation and Theory | 9 | What methodological orientation was stated to underpin the study? e.g.  grounded theory, discourse analysis, ethnography, phenomenology, and content analysis | Inductive and deductive thematic analysis | Methods – 6 |
| Participant selection | | | | |
| Sampling | 10 | How were participants selected? e.g. purposive, convenience, consecutive, snowball | Purposive selection | Methods – 5 |
| Method of approach | 11 | How were participants approached? e.g. face-to-face, telephone, mail, email | Face-to-face, telephone and email | Methods – 5 |
| Sample size | 12 | How many participants were in the study? | Total number of participants= 30 | Result - 6 |
| Non-participation | 13 | How many people refused to participate or dropped out? Reasons? | None | N/A |
| Setting | | | | |
| Setting of data collection | 14 | Where was the data collected? e.g. home, clinic, workplace | Online and face-to-face meetings at respective OPDs and DSOs in Dhaka and Chattogram | Methods – 5 |
| Presence of nonparticipants | 15 | Was anyone else present besides the participants and researchers? | We asked participants to be in a private place preferred by them where they would not be disturbed. | N/A |
| Description of sample | 16 | What are the important characteristics of the sample? e.g. demographic data, date | As reported in the result section | Results –5- 6 |
| Data collection | | | | |
| Interview guide | 17 | Were questions, prompts, and guides provided by the authors? Was it pilot tested? | All questions are provided in an additional file. | Appendix 1. B |
| Repeat interviews | 18 | Were repeat interviews carried out? If yes, how many? | There was no repeat interview. | N/A |
| Audio/visual recording | 19 | Did the research use audio or visual recording to collect the data? | All the interviews and FGDs both face-to-face and online were audio recorded | Methods –5 |
| Field notes | 20 | Were field notes made during and/or after the interview or focus group? | Other authors took notes during interviews and FGDs | Methods – 5 |
| Duration | 21 | What was the duration of the interviews or focus groups? | The interviews lasted for  40-50 minutes and FGDs lasted for 110-120 minutes on average | Methods – 5 |
| Data saturation | 22 | Was data saturation discussed? | Data saturation was reached when no new data emerged from the interviews. | Methods –5 |
| Transcripts returned | 23 | Were transcripts returned to participants for comment and/or correction? | No | N/A |
| Domain 3: analysis and findings | | | | |
| Data analysis | | | | |
| Number of data coders | 24 | How many data coders coded the data? | Three | Methods -5 |
| Description of the coding tree | 25 | Did the authors provide a description of the coding tree? | Yes | Table 3 |
| Derivation of themes | 26 | Were themes identified in advance or derived from the data? | Derived from the data | Methods -6 |
| Software | 27 | What software, if applicable, was used to manage the data? | No | NA |
| Participant checking | 28 | Did participants provide feedback on the findings? | No | N/A |
| Reporting | | | | |
| Quotations presented | 29 | Were participant quotations presented to illustrate the themes/findings?  Was each quotation identified? e.g. participant number | Yes | Results – 8- 13 |
| Data and findings consistent | 30 | Was there consistency between the data presented and the findings? | Yes | N/A |
| Clarity of major themes | 31 | Were major themes clearly presented in the findings? | Yes | Results –8-13Table 3 |
| Clarity of minor themes | 32 | Is there a description of diverse cases or discussion of minor themes? | Yes | Results – 8-13 |

**Appendix: D**

Highlights of the Results

**Theme 1: Acceptability and relevance of the PRT programme**

**Perspectives**

- The PRT programme was a social and inclusive training programme that paved the way for learning about MH and the rights of persons with disabilities *(PRT training was a capacity-building opportunity and empowering)*
- The learning environment of the PRT programme was very effective for the capacity building of peer responders (*Peer responders regularly attended training and supervision sessions. They participated in the DSO’s activities with enthusiasm*)

**Motivation for participating**

- Understanding MH was essential for themselves and also for other people with disabilities (*Peer responders deeply felt the need to understand their well-being and support other persons with disabilities*).
- To get a deeper understanding of disability
- To reduce negative perspectives about disability and MH.

**Acceptability of programme activities**

- Peer responders perceived training contents as relevant for learning the basics of MH conditions to support the community *(They found it interesting to learn about the unique issues of mental health through participatory training, roleplays, and group discussions)*
- Training also helped them identify severe MH issues such as schizophrenia, major depressive disorders, and obsessive-compulsive disorders
- Disability-related content reflected their personal feelings, provided them with a sense of social protection, and also created a way to learn about themselves *(Door-to-door community visits and courtyard meetings created a way for them to share their inner thoughts and made them feel supported)*

**Increased MH literacy of persons with disabilities**

- Increased understanding of MH and available MH services
- Helpful in eradicating the stigma associated with MH
- Positively influenced the MH-seeking behaviour of their target participants, and the demand for MH-care in the community echoes the statement's relevancy.
- To increase awareness of persons with disabilities/peers to identify their MH issues alongside their family members.

**Accessible MH support in the community**

- Need-based support was provided, such as primary counselling or referral, especially in rural areas where no service was available.
- It served a group of the disadvantaged population of the community

**Positive impact on the well-being of the peer responders:**

- Contributed to improving the well-being of the peer responders as it helped to increase their self-confidence and sense of fulfilment for contributing to the community.
- An important role of being a spokesperson for their peers who have disabilities

**Theme 2: Challenges and Barriers**

**Challenges of receiving PRT training**

- Training duration was somewhat inadequate to understand the overall content of the training module.
- No previous exposure to MH-related knowledge was a major barrier to understanding the content at first.
- Low socioeconomic and educational status, increased challenges in translating their acquired knowledge from the training. Contents were not appropriately socio-culturally customised, which could negatively impact their service delivery

**Challenges of providing PRT services:**

- Providing MH services to those with low MH literacy
- Stigma about MH
- Applying the psychometric assessment questionnaire and home visits for persons with visual impairment
- Demonstrating behavioural change communication material for peer responders with visual impairment
- Ensuring treatment adherence of participants

**Logistical Challenges:**

- Long walks and lack of convenient transportation options were among the primary issues
- Challenges in providing services during outbreaks or natural disasters
- Lack of financial support

**Theme 3: Recommendations for scaling-up**

**Training Programme:**

- Increasing the duration of the training session
- Including more practical activities to reduce monotony in the training module
- Advance training in improving the communication skills of peer responders
- MH training module should be inclusive and understandable for non-specialists
- Providing guidelines or checklists for providing proper referral
- Periodic monitoring and supervision to ensure higher-quality care
- Opportunity for refresher training

**Service delivery:**

- Including financial support for better sustainability
- Increasing the number of peer responders to ensure greater service availability
- Capacity building of peer responders as master trainers for providing future PRT programmes to other suitable candidates
- Getting formal recognition such as identity cards to be recognised as MH advocates at the community level
- Conducting/ arranging community awareness programmes before engaging peer responders to make people aware of available services.

**Integration and expansion:**

- Advocating for the integration of the PRT services into existing MH services
- Ensuring collaboration of governmental and non-governmental organisations to increase sustainable, easily accessible, and community-based inclusive MH services
- Expanding access to necessary psychiatric medications through the services of peer responders
- Conducting large-scale evidence-based training programmes to assess the effectiveness and build capacity of more persons with disabilities as peer responders
